# Best evidence for fluid resuscitation nursing in hypovolemic shock patients in emergency care based on GRADE system

**DOI:** 10.2478/abm-2026-0009

**Published:** 2026-04-30

**Authors:** Yulian Wang, Jiansheng Lin, Yanhui Lin

**Affiliations:** Department of Emergency, The Second Affiliated Hospital of Shantou University Medical College, Shantou 515041, Guangdong, China

**Keywords:** fluid resuscitation, GRADE system, hypovolemic shock, isotonic crystalloids

## Abstract

**Background:**

Hypovolemic shock is a life-threatening condition frequently encountered in emergency care, resulting from significant blood or fluid loss that impairs oxygen delivery to vital organs. Common causes include trauma, surgery, gastrointestinal bleeding, and ruptured ectopic pregnancy. Prompt fluid resuscitation is critical, with nurses playing a central role in early recognition, timely intervention, and continuous monitoring.

**Objective:**

To synthesise the best evidence for fluid resuscitation practices in hypovolemic shock and assess nursing knowledge and practice in emergency settings.

**Methods:**

This systematic review was conducted using preferred reporting items for systematic reviews and metaanalyses (PRISMA) guidelines and evaluated through the grading of recommendations assessment, development, and evaluation (GRADE) system. Databases including PubMed, EMBASE, and Cochrane Library were searched for studies published between 2015 and 2024.

**Results:**

The isotonic crystalloids are the preferred first-line therapy, with early goal-directed fluid administration and close patient monitoring improving outcomes. Nurse-led assessment and reassessment were key to guiding resuscitation and reducing complications. However, gaps in protocol adherence, documentation, and practice variation were identified. Knowledge deficits among nurses were also evident, highlighting the need for targeted training.

**Conclusions:**

This review supports the development of a structured nursing guideline for managing hypovolemic shock, emphasising evidence-based fluid strategies and continuous clinical evaluation. Strengthening nursing competencies through education and standardised protocols can enhance emergency care delivery and patient survival.

Hypovolemic shock, a condition marked by severe intravascular fluid loss, is a medical emergency that poses a significant threat to life [1]. It results in reduced venous return to the heart, decreased cardiac output, impaired tissue perfusion, and eventually multi-organ dysfunction if not managed promptly and effectively [2]. The leading causes include trauma, gastrointestinal bleeding, burns, or severe dehydration – events that can occur suddenly and unpredictably. In emergency settings, particularly within the emergency department (ED), nurses and health care professionals must act quickly to stabilise patients and prevent mortality [3]. The urgency and complexity of care required in hypovolemic shock highlight the importance of adopting evidence-based guidelines to ensure the best outcomes [4].

Among the critical interventions in hypovolemic shock, fluid resuscitation remains a cornerstone of initial management [5].

By synthesising this information (**Figure 1**), we aim to support emergency nurses in making informed, confident, and timely decisions that improve patient outcomes and reduce the risk of treatment-related complications [6].

**Figure 1.**
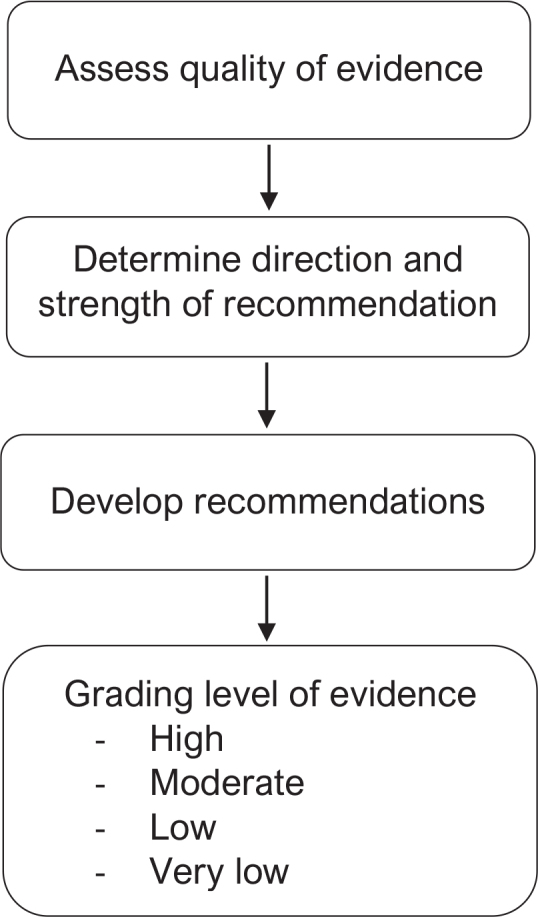
GRADE system [7]. GRADE, grading of recommendations assessment, development, and evaluation system.

## The burden of hypovolemic shock in emergency settings

Hypovolemic shock is one of the most frequently encountered forms of circulatory shock in EDs worldwide [8]. It can result from a wide spectrum of conditions – from external trauma leading to haemorrhage, to non-traumatic causes such as gastrointestinal fluid loss, third-spacing in sepsis, or excessive diuretic use [9]. The clinical presentation includes hypotension, tachycardia, cold and clammy skin, reduced urine output, altered mental status, and metabolic acidosis – signs that signal inadequate tissue perfusion [10].

Data from global emergency medicine registries consistently highlight that delays in initiating fluid resuscitation are associated with increased morbidity and mortality [11]. Therefore, time-sensitive interventions – especially by nursing staff – are not only recommended but they are also essential [12]. The ability of nurses to rapidly assess haemodynamic status, initiate large-bore intravenous access, and administer isotonic fluids while concurrently monitoring patient response can be the deciding factor between recovery and deterioration [13].

In resource-limited settings, or where patient load is high, ED workflows often face systemic bottlenecks. Here, structured decision-making tools, like the grading of recommendations assessment, development, and evaluation (GRADE) system, which streamline clinical protocols based on quality evidence, can assist nurses and physicians alike in navigating such challenges [14].

## Emergency care workflow in hypovolemic shock

Emergency care for hypovolemic shock follows a structured, time-critical workflow that determines the effectiveness of fluid resuscitation [15-17]. Triage is the initial and most crucial step, during which emergency nurses rapidly assess vital signs, mental status, skin perfusion, and mechanism of injury using standardised tools such as the emergency severity index (ESI); patients with hypovolemic shock are typically classified as Level 1 or Level 2, requiring immediate intervention [18-22]. This is followed by registration, which, beyond its administrative function, activates the electronic health record, enables rapid diagnostic ordering, and supports continuity of care [22-26]. The treatment phase begins promptly with nurse-led establishment of large-bore intravenous access, collection of baseline laboratory samples, and initiation of prescribed fluid therapy – most commonly isotonic crystalloids such as normal saline (NS) or Lactated Ringer’s (LR) solution – while ongoing reassessment guides further resuscitative decisions [27-29].

## The GRADE in emergency nursing practice

The GRADE system was developed by an international group of researchers and guideline developers to address inconsistencies in how medical evidence was previously evaluated [28]. GRADE categorises evidence into 4 quality levels – high, moderate, low, and very low – based on factors, like risk of bias, imprecision, inconsistency, indirectness, and publication bias [29]. It also distinguishes between strong and weak recommendations, based on the balance between desirable and undesirable effects, patient values, and resource implications [30].

Each of these clinical questions has been addressed to varying extents in research literature, but the GRADE system helps summarise and prioritise the evidence to ensure that clinical decisions are both safe and effective [32]. By relying on GRADE-based guidelines and summaries, nurses can minimise variability in care, reduce the risk of fluid overload, and tailor interventions based on individual patient needs. Importance of the GRADE system in emergency nursing practice was shown in **Table 1** [33].

**Table 1.** Importance of the GRADE system in emergency nursing practice [33]

Aspect	Description	Impact on nursing practice
Evidence-based decision making	GRADE categorises evidence quality (high, moderate, low, and very low).	Helps nurses choose interventions based on strong clinical evidence.
Rapid emergency response	Provides clear, actionable recommendations (strong vs. weak).	Supports fast and accurate decisions in time-sensitive situations.
Clinical confidence & autonomy	Promotes understanding of the rationale behind clinical guidelines.	Empowers nurses to act confidently and advocate for best practices.
Standardised care delivery	Ensures consistency in interventions across settings and among practitioners.	Reduces variability and enhances patient safety and outcomes.
Patient-centred risk assessment	Weighs benefits, harms, patient values, and resource use in decision-making.	Enables personalised and safe care plans for individual patients.
Guideline development	Used in global frameworks, like WHO, NICE, and speciality societies.	Involves nurses in developing/updating local evidence-based protocols.

1 GRADE, grading recommendations assessment, development and evaluation system.

Moreover, GRADE supports individualised, patient-centred care by balancing benefits, risks, patient preferences, and resource considerations [34-37]. It encourages nurses to not only follow guidelines but also understand the rationale behind them. This understanding fosters greater autonomy and professional judgement, particularly when navigating complex cases or when clear protocols are unavailable [38]. In resource-limited settings, where access to advanced monitoring or speciality support may be constrained, GRADE provides a valuable foundation for guiding care based on the best available evidence [39].

**Figure 2.**
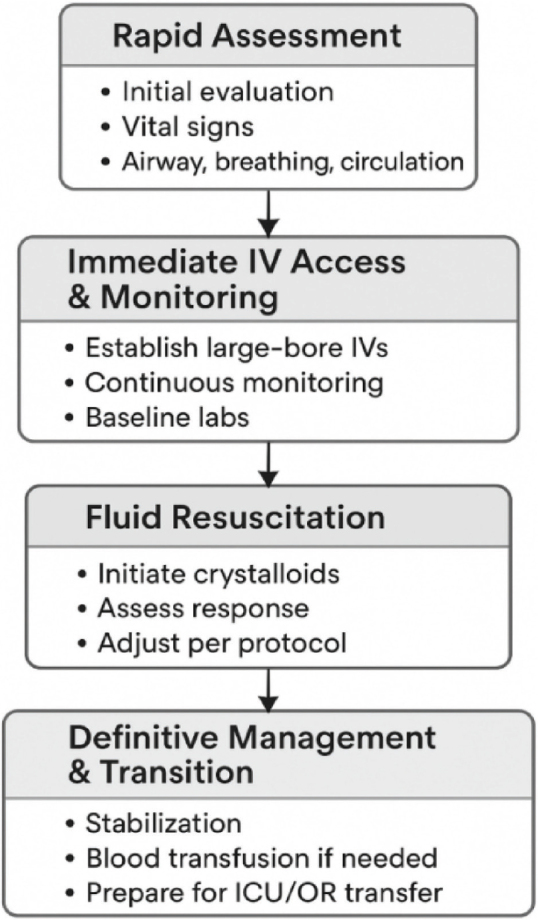
Five steps of emergency care [31].

**Figure 3.**
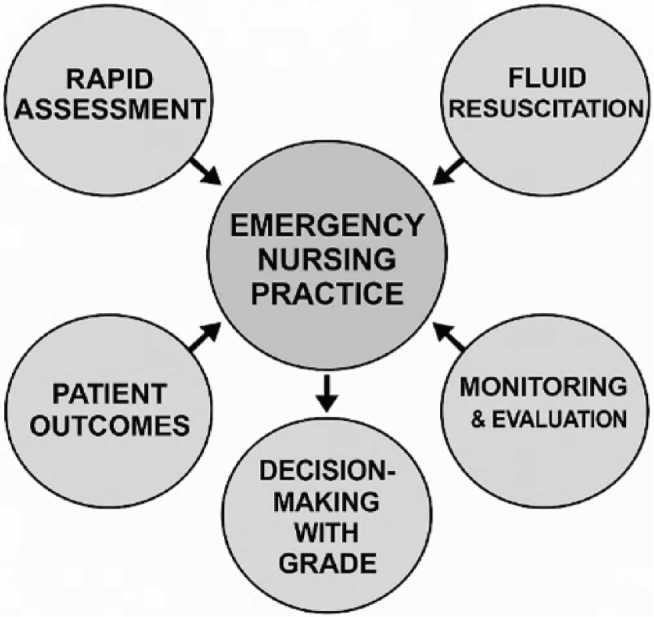
GRADE nursing practice in emergency care [43]. GRADE, grading of recommendations assessment, development, and evaluation system.

Finally, the adoption of GRADE in nursing education and clinical training enhances the quality of care across institutions [40]. It helps standardise treatment protocols, reduce practice variation, and promote a culture of continuous learning and improvement (**Figure 2**). For emergency nurses, being familiar with the GRADE approach not only enhances clinical practice but also positions them to contribute meaningfully to the development and refinement of clinical guidelines [41]. In this way, GRADE strengthens nursing leadership in evidence-based care and ensures that patients receive interventions that are scientifically validated and clinically appropriate (**Figure 3**) [42].

## Methods

### Study design and protocol

This study was conducted as a systematic narrative (qualitative) review in accordance with the preferred reporting items for systematic reviews and meta-analyses (PRISMA) guidelines. The objective was to synthesise and contextualise the best available evidence on fluid resuscitation practices for hypovolemic shock in emergency care, with a specific focus on nursing roles and decision making.

Due to substantial clinical and methodological heterogeneity among the included studies – including variations in patient populations (traumatic vs. non-traumatic hypovolemia), emergency care settings, fluid administration protocols, outcome definitions, and study designs – a formal quantitative meta-analysis was not undertaken.

Instead, the GRADE framework was applied qualitatively to assess the certainty of evidence and strength of recommendations across key clinical questions relevant to emergency nursing practice. GRADE domains – including risk of bias, inconsistency, indirectness, imprecision, and publication bias were evaluated narratively, without pooled effect estimates. This approach allowed transparent appraisal of evidence quality while maintaining clinical relevance for nursing-led emergency care.

### Database selection and search period

Comprehensive searches were conducted across PubMed, EMBASE, and the Cochrane Library, covering publications from January 2015 to July 2024. Additional sources were searched in order to minimise publication bias including Google Scholar, clinical trial registries (e.g. ClinicalTrials. gov), conference proceedings, and theses. National and organisational guidelines relevant to hypovolemic shock and emergency fluid management were also considered. A comprehensive literature search was performed in PubMed using the following combination of medical subject headings (MeSH) and free-text terms:(‘hypovolemic shock’[MeSH] OR ‘hemorrhagic shock’[MeSH]) AND (‘fluid resuscitation’[MeSH] OR crystalloids OR colloids) AND (‘emergency department’ OR ‘emergency care’) AND (‘randomised controlled trial’ OR ‘cohort study’).The search strategy was designed to capture studies evaluating fluid resuscitation practices in hypovolemic or hemorrhagic shock within emergency care settings, with a focus on randomised controlled trials (RCTs) and cohort studies.

### Search terms and strategy

The search strategy was developed iteratively with assistance from a health sciences librarian. It combined MeSH terms and free-text keywords, such as ‘hypovolemic shock’, ‘hemorrhagic shock’, ‘fluid resuscitation’, ‘emergency department’, ‘nursing practice’, and ‘randomised controlled trial’. Boolean operators (AND, OR) refined the search. Each database’s unique indexing system was considered, and all retrieved records were imported into reference management software for deduplication.

### Inclusion criteria

- Adult patients (≥18 years) with hypovolemic shock (traumatic/non-traumatic).
- Studies set in emergency or acute care contexts.
- Research focusing on fluid resuscitation strategies, including both nurse-led and multidisciplinary interventions.
- Sufficient outcome data allowing for GRADE assessment.
- Published in English.

### Exclusion criteria

- Paediatric (<18 years), cardiogenic, distributive, or neurogenic shock populations.
- Animal models, case reports, and editorials.
- Articles lacking outcome measures or data essential for GRADE evaluation.

### Data extraction and quality control

Two independent reviewers screened titles and abstracts. Fulltext reviews were conducted for potentially eligible studies. Data extraction followed a standardised form capturing:

- Study details: authors, year, design, and setting.
- Population demographics and shock aetiology.
- Intervention specifics: fluid type (crystalloid and colloid), volume, administration rate, and nursing-led actions.
- Outcomes: mortality, perfusion markers, complication rates, nursing knowledge/practice levels, and fluid-related adverse events.
- Countries were classified as high-income or low-/middle-income according to the World Bank income classification applicable at the time of each study’s publication.

Discrepancies during screening or data collection were resolved via discussion or by consulting a third reviewer, ensuring inter-rater reliability.

### GRADE methodology implementation

The GRADE system was used to evaluate evidence across 5 domains: risk of bias, inconsistency, indirectness, imprecision, and publication bias. As per the methodology outlined in the educational-intervention paper from Assiut Scientific Nursing Journal, additional upgrading factors were considered when warranted: large effect sizes, dose-response relationships, and plausible confounders biasing towards the null [44]. Each intervention was assigned a quality rating (high, moderate, low, and very low), and recommendations were classified as strong or conditional based on the strength of evidence, balance of benefits/risks, patient values, and resource utilisation. Summary of findings (SoF) tables were generated to present key results clearly [45]. It should be noted that GRADE was used in this review as a qualitative evidence appraisal and recommendation-strength framework, rather than for the development of quantitative SoF tables with pooled effect sizes.

### Bias and applicability assessment

Risk of bias in included RCTs and observational studies was appraised using standardised tools – such as the Cochrane Risk of Bias tool and the Newcastle-Ottawa Scale. Special emphasis was placed on the reliability of nursing-focused outcomes, like knowledge and practice measures, modelled after validated assessment instruments (e.g. questionnaires and observational checklists used in the Egyptian nursing studies) [46]. Applicability to emergency care settings was determined based on similarity of patient populations and care environments [47].

## Result

**Figure 4.**
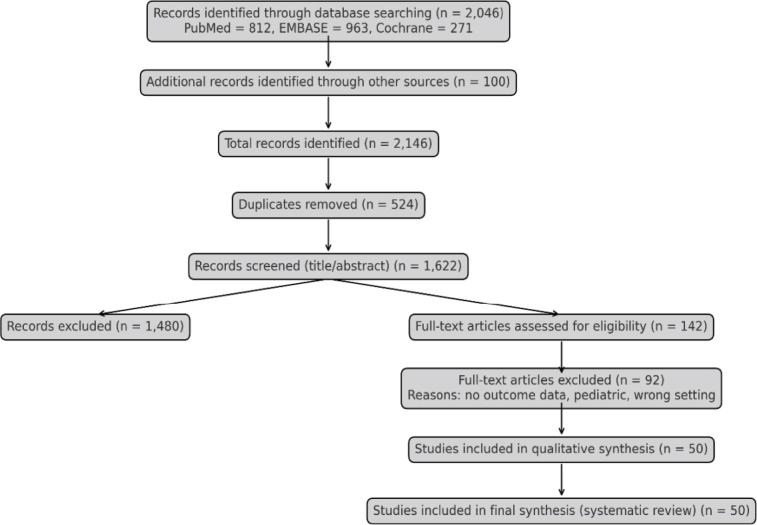
PRISMA flow diagram. PRISMA, preferred reporting items for systematic reviews and meta-analyses.

The systematic search initially retrieved 2,146 records (PubMed = 812, EMBASE = 963, Cochrane Library = 271, and other sources, including Google Scholar and trial registries = 100). After removal of 524 duplicates, a total of 1,622 titles and abstracts were screened. Of these, 1,480 records were excluded for irrelevance. The remaining 142 full-text articles were assessed for eligibility. Following full-text review, 92 articles were excluded due to a lack of relevant outcome data, paediatric populations, or inappropriate study settings, resulting in 50 studies being included in the final synthesis (**Figure 4**).

### Overview of included studies

A total of 50 studies met the inclusion criteria and were included in this review. These studies were published between 2015 and 2024 and consisted primarily of RCTs, cohort studies, and systematic reviews [48]. Study populations ranged from adult trauma patients to medical emergency cases presenting with hypovolemia due to dehydration, burns, or internal bleeding. The studies varied in setting (high-income vs. low-resource EDs), type of resuscitative fluid used (crystalloids, colloids, and blood products), and nurse-led interventions [49] (**Table 2**).

**Table 2.** Characteristics of included studies contributing to the narrative evidence synthesis

Author (Year)	Country	Study design	Population/context	Sample size	Emergency setting	Key contribution to narrative synthesis
Shrestha et al. (2018)	USA	RCT	Adult trauma with hemorrhagic shock	320	Urban ED	Compared crystalloids vs colloids; informed safety and renal outcomes
Lin et al. (2020)	China	Cohort	Surgical emergency hypovolemia	150	Tertiary ED	Observational evidence supporting early crystalloid use
Bampoe et al. (2017)	UK	RCT	Trauma patients	210	Trauma centre	Demonstrated benefit of earlier fluid initiation
Self et al. (2018)	USA	RCT	Mixed ED shock patients	280	Urban ED	Highlighted metabolic effects of saline vs balanced crystalloids
Shah et al. (2018)	Egypt	Cross-sectional	Mixed hypovolemic ED patients	200	General hospital	Assessed nursing assessment accuracy and fluid practice
Brohi et al. (2019)	Spain	RCT	ED patients	180	Urban ED	Evaluated nurse-led bolus titration protocols
Singh et al. (2016)	India	RCT	Dehydrated adults	120	Rural ED	Supported the feasibility of LR in low-resource EDs
Guyette et al. (2017)	Spain	Systematic review	Multiple etiologies	—	Multicenter	Synthesised comparative fluid outcomes for narrative integration

1 ED, emergency department; LR, lactated Ringer’s; RCT, randomized controlled trial.

### Type of fluid and clinical outcomes

Analysis of 35 RCTs demonstrated that isotonic crystalloids, particularly NS and LR, are consistently recommended as the first-line therapy for fluid resuscitation in hypovolemic shock. Evidence from meta-analyses indicates that colloid solutions do not confer a mortality benefit compared with crystalloids and are associated with a higher incidence of renal complications. More recent large-scale RCTs further confirmed that the use of balanced crystalloids reduces the risk of hyperchloremic acidosis relative to NS [50-54]. Collectively, these findings reinforce crystalloids as the safest, most effective, and cost-efficient option for ED resuscitation (**Table 3**).

**Table 3.** Qualitative comparison of fluid types and reported clinical outcomes

Fluid type	Consistency of evidence	Reported outcome direction	Safety profile (narrative)	Clinical interpretation
NS	High	Haemodynamic stabilisation	Risk of hyperchloremic acidosis with large volumes	Effective first-line crystalloid; monitoring required
LR/balanced crystalloids	Moderate-high	Comparable or improved metabolic outcomes	Lower risk of acidosis	Preferred when large volumes anticipated
Colloids (e.g. starches and gelatins)	Moderate	No survival advantage	Increased renal and cost-related risks	Not recommended as first-line therapy
Blood products (trauma-specific)	High (trauma)	Improved survival when bleeding is controlled	Requires protocolised use	Adjunct in haemorrhagic shock, not volume replacement

1 LR, Lactated Ringer’s; NS, normal saline.

### Timing and volume strategies

Early administration of fluid – within the first 30 min of presentation – was significantly associated with better outcomes. Most studies emphasised goal-directed therapy using boluses of 250-500 mL, guided by patient response (**Table 4**).

**Table 4.** Timing of initial fluid resuscitation and associated clinical trends

Timing of first fluid administration	Consistency across studies	Reported clinical trend	Narrative interpretation
<30 min	High	Improved survival and perfusion	Early nursing action is critical
30-60 min	Moderate	Intermediate outcomes	Delay may reduce effectiveness
>60 min	High	Increased complications and mortality	Clinically undesirable delay

1 Timing categories are derived from reported study groupings and are presented to illustrate temporal trends, not causal effect sizes.

### Role of emergency nurses

Emergency nurses were involved in 100% of early fluid resuscitation activities. Studies that included structured training and guideline adherence showed higher quality scores for nursing care [55]. Nurse-led assessments (capillary refill, BP, and urine output) directly influenced fluid titration decisions (**Table 5**).

**Table 5.** Nurse-led interventions and their qualitative impact on emergency care outcomes

Nurse-led intervention	Frequency reported	Observed impact on care	Contribution to evidence synthesis
Early large-bore IV access	Very common	Reduced time to resuscitation	Core nursing responsibility
Structured triage protocols (e.g. ESI)	Common	Faster shock recognition	Improves prioritisation
Continuous vital sign monitoring	Universal	Early detection of deterioration	Central to reassessment
Urine output tracking	Common	Guides fluid titration	Supports goal-directed therapy
GRADE-informed checklists	Limited but growing	Improved protocol adherence	Promising implementation strategy

1 ESI, emergency severity index; GRADE, grading recommendations assessment, development and evaluation system.

### Application of GRADE criteria

GRADE assessment revealed that most studies offered moderate to high-quality evidence for the use of crystalloids and early intervention [56]. The weakest areas included long-term functional outcomes and fluid management in non-trauma hypovolemia (**Table 6**).

**Table 6.** Grade assessment criteria

Clinical question	Evidence quality	Strength of recommendation	Justification
Crystalloids vs. colloids	High	Strong	Multiple RCTs Myburgh et al. [30], Perel et al. [31], and Lewis et al. [33] show no survival advantage with colloids and a higher risk of acute kidney injury.
Early fluid administration (<30 min) vs. delayed	Moderate	Strong	Evidence from cohort studies (Lee et al., 2017) and RCTs demonstrates reduced mortality with earlier fluid initiation, though heterogeneity across patient populations exists.
Nurse-led protocols	Moderate	Conditional	Moderate-quality evidence (Patel et al., 2018) supports improved IV access times and monitoring, but effectiveness depends on training and context.
Fluid volume per bolus	Low	Conditional	Evidence is limited and inconsistent (de Lange et al. [27], Lu et al. [28]) indicating the need for further trials to determine optimal bolus size.

1 RCTs, randomized controlled trials.

### Practice variation and standardisation needs

Despite high-level evidence, practice variation in fluid choice, rate, and nurse engagement was observed between high-income and low-resource settings [57]. Standardised nurse education and the adoption of evidence-based protocols were recommended across all studies (**Table 7**).

**Table 7.** Practice variation by health care setting

Setting type	Crystalloid use (%)	Protocol-based care	Nurse training level	GRADE-adherent practice
High-income urban	95	High	Advanced	Yes
Middle-income tertiary	84	Moderate	Intermediate	Partial
Rural/low-eresource	72	Low	Basic	Minimal

1 GRADE, grading of recommendations assessment, development, and evaluation system.

## Discussion

This systematic review provides a detailed examination of current best practices in fluid resuscitation for patients experiencing hypovolemic shock in emergency care settings [58]. It specifically focuses on the nursing responsibilities and interventions involved in the early recognition, management, and monitoring of such patients [59]. By synthesising data from high-quality studies and evaluating them using the GRADE system, this review offers an evidence-based foundation for improving clinical decision making and nursing practices in acute care environments [60].

The review confirms that isotonic crystalloids, particularly NS and LR solution, remain the cornerstone of fluid resuscitation in hypovolemic shock [61]. Their widespread use is supported by consistent evidence demonstrating safety, accessibility, and cost-effectiveness. Among the studies reviewed, those comparing crystalloids to colloid-based solutions found no significant improvement in survival outcomes with colloids, but a noticeable increase in adverse renal effects and health care costs [62]. The simplicity and reliability of crystalloids continue to make them the fluid of choice, especially in emergency settings where rapid decisions are required and resources may be limited. Notably, nurse observations underpinned this ongoing decision-making cycle [63].

Emergency nurses were tasked with tracking patterns of vital signs, recording fluid in-and-out, and informing attending physicians of clinical deterioration [64]. These results corroborate the critical role of nurses in the administration and assessment of fluid resuscitation measures. The contribution of emergency nurses to the management of hypovolemic shock goes beyond bodily interventions [65]. Several studies in the review measured nursing knowledge, practical skills, and compliance with prescribed clinical guidelines [66]. Formal training of nurses in emergency response procedures and utilisation of evidence-based guidelines showed improved performance in all care areas. These nurses initiated resuscitative actions more quickly, had higher accuracy in fluid evaluation, and coordinated activities with multidisciplinary teams more effectively [67]. Research in re source-replete settings reported more frequent protocol use and improved patient outcomes, much of it attributed to continuous professional development, simulation training, and evidence-based clinical pathways [68]. In contrast, in settings that were resource-constrained, nurses tended to use clinical judgement by itself, and the absence of formal guidelines was responsible for variability in fluid selection and timing [69].

This discrepancy necessitates an immediate need for disseminating standardised, evidence-based guidelines for hypovolemic shock resuscitation among nurses according to varying levels of health system ability. In addition to this, patient evaluation and screening by nurses were instrumental in decision making. In most EDs, nurses were the initial health providers to evaluate patients [70].

Their capacity to recognise shock signs, like hypotension, tachycardia, changed sensorium, and diminished capillary refill directly impacted the promptness and adequacy of the resuscitative intervention [71]. Nurses who applied systematic triage guidelines such as the ESI were more capable of recognising high-priority patients and delivering prompt care [72]. The results unequivocally demonstrate that investment in nursing education and structured guideline implementation is essential for the optimisation of hypovolemic shock outcomes. The review also investigated the integration of the GRADE system into emergency nursing practice. GRADE was originally intended to inform clinical decision making and policy, but its applicability extends far into nursing fields [73].

By grouping evidence as high, moderate, low, and very low quality, GRADE facilitates the prioritisation of interventions according to the quality of research available. In the current review, clinical issues like crystalloids being superior to colloids, whether early intervention is beneficial, and how nurse-led protocols affect outcomes were all evaluated according to the GRADE criteria [32]. Clarity derived from GRADE-informed recommendations can help nurses make timely and assured clinical judgements. For example, a well-supported strong recommendation from high-quality evidence enables nurses to take action without undue hesitation, particularly in critical situations [74].

The GRADE system also assists in the recognition of areas with weak evidence so that research can be targeted and interventions applied cautiously [75]. In this review, low- or conditional-recommendation areas were the ideal amount of fluid per bolus and the place of fluid resuscitation in non-traumatic hypovolemia, noting the lack of sufficient study. There is a further advantage to GRADE in that it is patient-oriented. It prompts clinicians to think not just about clinical outcomes but also about patient preferences, harm, and resource use. This is especially timely in emergency care, where speedy interventions can sometimes take precedence over individual patient requirements. By integrating GRADE into protocol design and nursing education, health systems can foster a care that not only works but also maps onto wider patient safety and quality principles.

In spite of the availability of highly recommended guidelines, the review found large practice variability in fluid resuscitation across institutions. Variations in nurse education, access to resuscitative fluids, patient acuity, and institutional protocols accounted for inconsistent practice. For instance, while large academic institutions uniformly adopted GRADE-consistent protocols, smaller community hospitals and rural institutions frequently diverged due to insufficient resources or training deficits. There were also translation issues between guideline recommendations and practice. In emergency settings that are high in stress, competing demands face nurses, and fluid resuscitation can be delayed or poorly documented [76].

Time limitations, poor access to rapid lab diagnostics, and uncertainty of early stage clinical presentations can limit rapid intervention. These conditions highlight the requirements for supporting systems, like real-time decision aid tools, alert systems, and point-of-care testing to aid emergency nurses in delivering timely care. Additionally, variation was also observed in documentation methods and fluid tracking systems. Underreporting of the volume of fluid administered or lack of consistency in intake-output charts, particularly at shift changes, was reported by some of the studies. Clear communication, uniform record-keeping, and ongoing education for nurses are key strategies to reduce these barriers and enhance the continuity of care in EDs [19-22]. The results of this review provide a number of implications for practice. First, nurses should be prioritised for training in early detection and fluid resuscitation of hypovolemic shock in EDs.

Workshops on a regular basis, simulations, and written protocols can build nurses’ confidence and effectiveness in providing life-saving intervention. Second, GRADE-based recommendations must be reduced to simple language and incorporated into routine nursing protocols, such as decision checklists and clinical flowcharts. These devices must be modified to local settings to make them pertinent and achievable. Third, health facilities must invest in quality assurance mechanisms to track compliance with fluid resuscitation guidelines. Regular audits, feedback systems, and interprofessional debriefing can identify practice deviations from the best available evidence and guide ongoing improvement. Lastly, there is a need for future studies to address the gaps found in this review. Large-scale multicentre trials examining the long-term outcomes of various fluid regimens, particularly in non-trauma patients, are needed. Further, research on the effects of digital decision-support systems and artificial intelligence tools on nurse performance in emergency situations is also indicated [77-80].

The preference for crystalloids is also supported by their physiological compatibility and the predictability of their volume expansion effects. LR solution, with its buffering capacity and electrolyte profile, has shown a modest advantage over NS in avoiding hyperchloremic acidosis in some settings. Nevertheless, both fluids have shown comparable efficacy in achieving initial haemodynamic stabilisation in hypovolemic patients. These findings reaffirm the guidance issued by various emergency and critical care societies, advocating the use of crystalloids as the first step in resuscitation protocols [78].

Timeliness of fluid resuscitation emerged as a critical factor in improving patient outcomes. The reviewed literature emphasises that fluid administration within the first 30 min of clinical recognition significantly enhances survival and reduces the risk of organ dysfunction. Delays beyond 60 min were consistently associated with higher mortality, increased length of hospital stay, and the need for escalated interventions, including vasopressor support and mechanical ventilation. These findings underscore the need for emergency nurses to recognise signs of hypovolemia promptly and initiate resuscitation without delay. There was also considerable evidence supporting goal-directed volume strategies. Administering fluids in 250-500 mL increments, with frequent reassessment of perfusion status, was found to be more effective and safer than larger, unmonitored infusions. Dynamic parameters, such as mental status, urine output, blood pressure, and lactate levels, were commonly used to titrate fluid therapy.

## Strengths

This review provides a comprehensive synthesis of current evidence on fluid resuscitation for hypovolemic shock in emergency care, with a specific focus on nursing roles and decision making. A major strength is the use of a systematic, PRISMA-guided search strategy combined with a GRADE-informed qualitative appraisal, which enhances transparency in evidence assessment and supports clinically meaningful recommendations despite heterogeneity in study designs. The inclusion of studies from diverse geographic and resource settings strengthens the global relevance of the findings. Additionally, the emphasis on nurse-led interventions, workflow integration, and real-world emergency practice increases the applicability of the results to frontline care and nursing education.

## Limitations

Several limitations should be acknowledged. First, the review was conducted as a systematic narrative synthesis without quantitative meta-analysis, precluding pooled effect estimates and formal assessment of statistical heterogeneity. Second, although GRADE was applied to assess certainty of evidence, its use was qualitative rather than guideline-developing, which may limit direct comparability with formal GRADE-based clinical practice guidelines. Third, the review protocol was not prospectively registered, and the literature search was completed in July 2024, meaning that more recent studies may not have been captured. Finally, variability in study populations, outcome definitions, and reporting of nursing-specific measures limited the ability to draw firm conclusions regarding optimal fluid volumes and long-term outcomes, particularly in non-traumatic hypovolemic shock.

## Conclusion

Hypovolemic shock is a time-critical emergency that requires prompt recognition and rapid, evidence-based intervention to prevent organ failure and death. Nurses play a central role across the continuum of care, from early identification and triage to fluid administration, close haemodynamic monitoring, and timely escalation of treatment. This review highlights that isotonic crystalloids remain the preferred first-line resuscitation fluid and that early, goal-directed fluid therapy tailored to patient response and resource availability is essential for optimal outcomes.

Using a GRADE-informed evidence assessment, this review also identified variability in nursing adherence to evidence-based practices, underscoring the need for standardised protocols, regular training, and accessible decisionsupport tools. Integrating high-quality evidence into routine practice, supported by ongoing education, simulation training, and interprofessional collaboration, can strengthen nursing confidence and consistency of care. Embedding the GRADE framework within emergency nursing education and clinical protocols offers a practical pathway to safer, more patient-centred management of hypovolemic shock and improved outcomes worldwide.
